# How Melittin Inserts into Cell Membrane: Conformational Changes, Inter-Peptide Cooperation, and Disturbance on the Membrane

**DOI:** 10.3390/molecules24091775

**Published:** 2019-05-07

**Authors:** Jiajia Hong, Xuemei Lu, Zhixiong Deng, Shufeng Xiao, Bing Yuan, Kai Yang

**Affiliations:** Center for Soft Condensed Matter Physics and Interdisciplinary Research & School of Physical Science and Technology, Soochow University, Suzhou 215006, China; 20154208015@stu.suda.edu.cn (J.H.); 20174008027@stu.suda.edu.cn (X.L.); 20164208041@stu.suda.edu.cn (Z.D.); 20164208060@stu.suda.edu.cn (S.X.)

**Keywords:** antimicrobial peptide, melittin, cell membrane, membrane insertion, pore formation, computer simulations

## Abstract

Antimicrobial peptides (AMPs), as a key component of the immune defense systems of organisms, are a promising solution to the serious threat of drug-resistant bacteria to public health. As one of the most representative and extensively studied AMPs, melittin has exceptional broad-spectrum activities against microorganisms, including both Gram-positive and Gram-negative bacteria. Unfortunately, the action mechanism of melittin with bacterial membranes, especially the underlying physics of peptide-induced membrane poration behaviors, is still poorly understood, which hampers efforts to develop melittin-based drugs or agents for clinical applications. In this mini-review, we focus on recent advances with respect to the membrane insertion behavior of melittin mostly from a computational aspect. Membrane insertion is a prerequisite and key step for forming transmembrane pores and bacterial killing by melittin, whose occurrence is based on overcoming a high free-energy barrier during the transition of melittin molecules from a membrane surface-binding state to a transmembrane-inserting state. Here, intriguing simulation results on such transition are highlighted from both kinetic and thermodynamic aspects. The conformational changes and inter-peptide cooperation of melittin molecules, as well as melittin-induced disturbances to membrane structure, such as deformation and lipid extraction, are regarded as key factors influencing the insertion of peptides into membranes. The associated intermediate states in peptide conformations, lipid arrangements, membrane structure, and mechanical properties during this process are specifically discussed. Finally, potential strategies for enhancing the poration ability and improving the antimicrobial performance of AMPs are included as well.

## 1. Introduction

Antimicrobial peptides (AMPs) have been attracting more attention than ever due to the increasingly devastating threat of drug-resistant bacteria and superbugs to global health [1,2,3,4]. As reported by the World Health Organization (WHO), about 700,000 people are killed worldwide each year due to antibiotic resistance, and this number may rise to 10 million by 2050 if no effective action is taken [1]. Therefore, it is very urgent to develop new antimicrobial drugs. 

As a key component of the immune defense system of all classes of organisms, including animals, plants, and microbes [5,6], AMPs work as the first line of host defense against invading microbes [7]. Currently, more than 2500 types of AMPs have been identified and classified [8,9]. Although these peptides differ from each other notably in their primary and secondary structures, they have some crucial features in common [5,7]. For example, most AMPs are amphipathic and cationic and have exceptional broad-spectrum activities against microorganisms, including both Gram-positive and Gram-negative bacteria [5]. Usually, the target site of AMPs is microbial membranes, and their actions are mostly associated with membrane permeabilization via poration or disruption [6]. Note that cell membrane is regarded as the “Achilles heel” of bacteria. It is one of the most conservative parts of bacteria and has shown few changes during evolution [6,10]. Although the recent increasing use of certain AMPs (e.g., polymyxin B and colistin) against Gram-negative bacteria has induced resistance by some bacteria via outer membrane modification (e.g., *S. typhimurium* and *A. baumannii*) [11,12], AMPs still promise a fundamental solution to the serious threat of drug-resistant bacteria [4,7,10], and some are even considered to be the last defense line against Gram-negative superbugs [13]. Unfortunately, natural AMPs are generally not suitable for direct clinical use because of their poor cell penetration efficiency, high cytotoxicity, high cost, etc. [7,10]. As of today, only a few AMPs have been approved by the Food and Drug Administration [14]. Therefore, the development and even de novo design of novel peptides or peptide-based agents on the basis of the available AMPs are crucial [15,16,17,18,19]. However, a key obstacle to these efforts is the lack of detailed knowledge of the membrane action mechanism of AMPs at the molecular level, although extensive efforts have been made to research this issue for almost four decades [2,5,17]. What these peptides actually do on/in the membrane is still a mystery for scientists.

**Figure 1:**
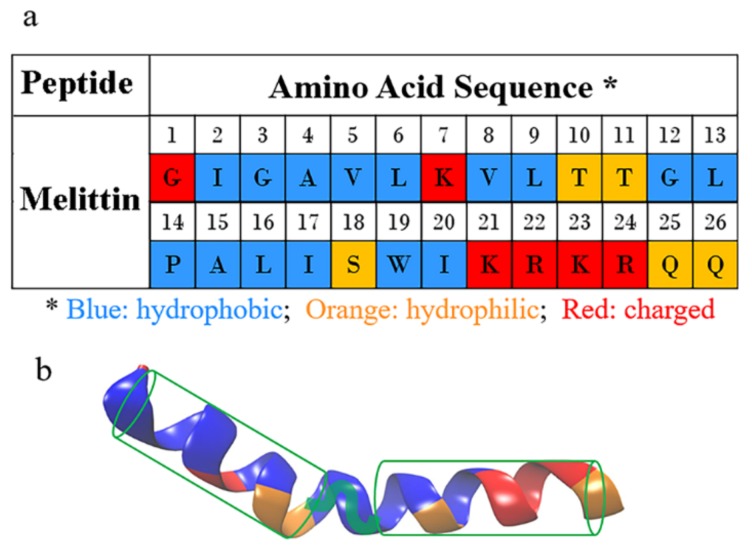
Structure of melittin. (**a**) The amino acid sequence of melittin. Different types of residues are colored differently: blue for hydrophobic residues, orange for hydrophilic ones, and red for charged ones; (**b**) the secondary structure of melittin showing the amphipathic α-helical configuration (when binding on a membrane). The two α-helixes of melittin are sketched with green cylinders, being connected with the kink part between them. Corresponding amino acid residues are highlighted with the same color scheme as in (**a**).

Peptide melittin is one of the most extensively studied AMPs. It was discovered sometime around 1970 as a major component in the venom of European honeybee *Apis mellifera* [20,21]. Melittin has demonstrated broad-spectrum bactericidal activity against both reference and clinical strains of bacteria [20,22,23] and even against antibiotic-resistant bacteria, such as *A. baumannii* and *P. Aeruginosa* [24,25]. Thus, it has been extensively used as a positive control to evaluate the antimicrobial activity of any newly discovered/developed AMP [26,27,28]. Melittin is a linear cationic peptide composed of 26 amino acid residues (see Figure 1a, (+)Gly-Ile-Gly-Ala-Val^5^-Leu-Lys(+)-Val-Leu-Thr^10^-Thr-Gly-Leu-Pro-Ala^15^-Leu-Ile-Ser-Trp-Ile^20^-Lys(+)-Arg(+)-Lys(+)-Arg(+)-Gln^25^-Gln-NH_2_), with a net charge of +6 under physiological pH conditions [5,29]. Thus, the amino acid residues close to its N- and C-terminal regions are mainly hydrophobic and hydrophilic, respectively [20]. As demonstrated by experiments and molecular dynamics (MD) simulations [30,31], after binding on a lipid membrane, melittin presents a bent, rod-like conformation with two α-helical parts connected by a non α-helical kink region. The polar and nonpolar residues distribute roughly symmetrically on the two sides of each helix, leading to the formation of an amphiphilic molecular configuration (Figure 1b). Such a cationic and amphipathic structure, which is regarded as the most characteristic configuration of AMPs, makes melittin one of the most representative models for understanding the mechanism of membrane permeabilization by AMPs [20,32,33,34,35].

Currently, the interaction mechanism of melittin with cell membranes is poorly understood, probably due to the small size of this peptide and the complexity of the lipid membrane. A well- accepted two-state model [29,36] of AMP action was first put forward by H.W. Huang. In this model, peptides bind on the membrane surface, followed by transmembrane insertion and the formation of pores. More efforts were further made to understand the molecular mechanism of melittin interaction with cell membrane by both experimental and simulation methods [32,37,38]. In general, it is accepted that the interaction can be divided into two stages: membrane binding and accumulation of peptides, followed by reorientation and insertion of peptides into membranes leading to the final formation of transmembrane pores. The characteristics of the transmembrane pores produced by melittin could be described with the barrel-stave or toroidal model. By using oriented circular dichroism (OCD) and neutron scattering, Huang et al. found that the properties of melittin-induced pores are similar to those of pores predicted by the toroidal model [29,39]. That is, along the formed pore, melittin molecules are vertically inserted into the lipid bilayer and the lipid monolayer bends continuously from the top to the bottom in a toroidal shape, so that the water core is lined by both melittin molecules and the headgroups of lipids. Moreover, Marrink and co-workers speculated that, besides the inserted melittin, other peptides may also line the mouth of the pore to stabilize its curvature [40]. In general, previous investigations indicate that the transition of peptides from the membrane surface-binding state (so-called *S* state) to the transmembrane-inserting state (so-called *T* state) is a key step for the formation of transmembrane pores. Such transition is influenced by several factors, such as peptide structure, concentration, and lipid composition. Among these factors, peptide concentration is the most important. It has been proven that the transition occurs only when the peptide-to-lipid ratio, P/L, is higher than a threshold value [32,37,41]. Furthermore, the P/L threshold is dependent on lipid composition [38]. For example, for a binary membrane system composed of 1,2-dioleoyl-sn-glycero-3-phosphocholine (DOPC) and 1,2-dioleoyl-sn-glycero-3-phospho-(1’-rac-glycerol) (DOPG), the threshold is approximately 1/45 [32,37]. It was assumed that a higher concentration of peptides could induce a stronger disturbance to the membrane structure (e.g., thinning and stretching), which would significantly decrease the high free-energy barrier for reorientation of peptides and facilitate their insertion [29,37,38]. Unfortunately, the exact driving forces for such a phase transition of peptides and the underlying mechanism of the transition remain largely unknown to date. Moreover, the actual melittin–membrane interaction might be more complicated than what it was previously proposed [40].

Various experimental and simulation methods have been employed to investigate the molecular details of the peptide–membrane interactions. NMR spectroscopy is one of the most powerful techniques to gain atomistic details of interactions between membrane-active peptides and cells [42,43,44,45,46,47]. For example, with solid-state NMR, Norisada et al. showed that melittin bound to a 1,2-Dimyristoyl-sn-glycero-3-phosphoglycerol (DMPG) membrane adopted a bent α-helical structure, with tilt angles of −32 and +30° for the N- and C-terminal helices, respectively; in contrast, melittin molecules in the transmembrane state had a pseudo-transmembrane bent α-helix [47]. OCD has also been widely applied to determine the conformation changes of peptides [31,48,49,50], while X-ray diffraction (XRD) could provide structural information relating to the membranes under the action of peptides and even the structure of transmembrane pores formed [32,37,51,52,53]. Although much progress has been made, it is still very difficult to experimentally acquire a direct observation of the membrane insertion process of a melittin molecule [54]. On the other hand, with their fast development in the past decades, computer simulations have been an excellent complementary tool to the experimental methods [55,56,57,58,59,60,61]. This so-called computational ‘microscopy’ technique is able to capture the interplay between lipids and proteins at a spatiotemporal resolution that is unmatched by other methods. It even allows us to zoom out from individual atoms and molecules to supramolecular complexes simultaneously with various length scales [62,63]. Here, in this mini-review, the recent advances in understanding the membrane insertion of peptides and the related influential factors are discussed from a computational view, from both kinetics and thermodynamics perspectives.

## 2. Kinetic Membrane Insertion Process of Melittin: Peptide Aggregation and Deformation; Membrane Disturbance Induced by Lipid–Peptide Interaction 

### 2.1. Simulation Models for Realization of Membrane Insertion of Melittin

As mentioned above, computer simulations have been considered as an attractive tool to study the molecular mechanism of the membrane insertion process of melittin [64,65,66,67,68]. However, we must be keenly aware that, for computer simulations, to realize the complete reorientation process of melittin from a membrane-binding state to a transmembrane state is not an easy task. Even with coarse-grained (CG) models, which are generally employed to construct systems with much larger spatial and temporal scales over all-atom (AA) simulations [58,69,70,71,72,73,74], it is almost impossible to observe the membrane insertion process of melittin in a time order of $10^{1}\;\mu s$ under the normal attacking conditions of the peptide. Thus, various “tricks” are generally used to accelerate the state transition velocity of melittin. For example, initially, some peptides might be partly embedded in the lipid membranes or placed directly inside the membrane with a pseudo-transmembrane orientation right before the simulations [75]. Moreover, the peptides might be introduced into both sides of the membrane at the beginning [76]. Such an initial configuration of peptide–membrane interaction was supposed to be able to avoid the potential artificial membrane stress induced by the finite-size effect [54,76,77,78]. Furthermore, thinner bilayer membranes composed of short-tail lipids (e.g., 1,2-dilauroyl-sn-glycero-3-phosphocholine, DLPC) have also been widely used to increase the sampling possibilities of peptides inside the membrane and to reduce the possible free-energy barrier for realizing melittin reorientation behavior [78,79].

### 2.2. Peptide-to-Lipid Ratio Dependence

**Figure 2:**
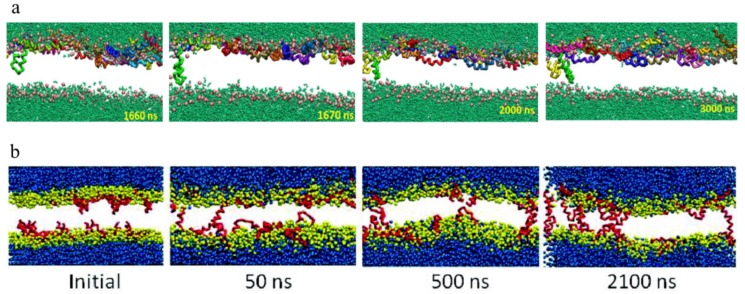
Two successful examples showing the membrane insertion process of melittin with different initial simulation conditions. (**a**) Transmembrane penetration of melittin into a DPPC/POPG membrane. P/L~1/21. Initially, peptides are adsorbed on the outer surface of the membrane. After 1600 ns, one U-shaped melittin penetrates into the bilayer and soon reaches the inner leaflet at 1670 ns. After that, aggregation occurs between the first and a second U-shaped peptide. Such aggregation further stabilizes the penetration state of melittin (e.g., 2000~3000 ns). Here, the peptides are colored differently for ease of understanding. For lipids, the PO_4_ groups are drawn in pink; while tails are not shown for clarity. Polarizable water is depicted in green. The figure is adapted with permission from [75], copyright 2013, American Chemical Society. (**b**) Translocation of melittin across a DPPC lipid bilayer at P/L~1/21. Initially, melittin peptides are adsorbed on both sides of the membrane. Some of the peptides insert into the membrane quickly (at 50 ns) in a U-shaped configuration. Then, multiple aggregates of peptides occur in the membrane interior (at 500 ns), and finally, transmembrane pores are formed (~2100 ns). Peptides are shown only by protein backbones (red). CG water molecules are shown as blue beads, while lipid headgroups as yellow beads. The figure is reprinted with permission from [76], copyright 2012, American Chemical Society.

Figure 2 shows two successful examples of the membrane insertion process of melittin obtained in simulations. Although these two events were totally different with respect to the initial conditions, including the peptide locations, attacking manners of peptides (from one side vs. two sides), and even the lipid environments (DPPC (1,2-dipalmitoyl-sn-glycero-3-phosphocholine)/POPG (1-palmitoyl-2-oleoyl-sn-glycero-3-phosphatidylglycerol) bilayer vs. DPPC bilayer), the membrane insertion process of melittin demonstrates many common features. Specifically, the occurrence of state transition of melittin, i.e., from surface binding to transmembrane insertion, is determined by the peptide-to-lipid ratio, i.e., P/L. As shown in simulations [40,78], the reorientation of melittin and its membrane insertion appeared only when P/L ≥ 1/43. Moreover, with a further increase of P/L (e.g., ~1/21), the insertion of melittin occurred more frequently [75,76]. This dependence of melittin insertion on peptide concentration is in good agreement with the experimental observations [32,37].

### 2.3. Inter-Peptide Association Based on Local Accumulation of Peptides

**Figure 3:**
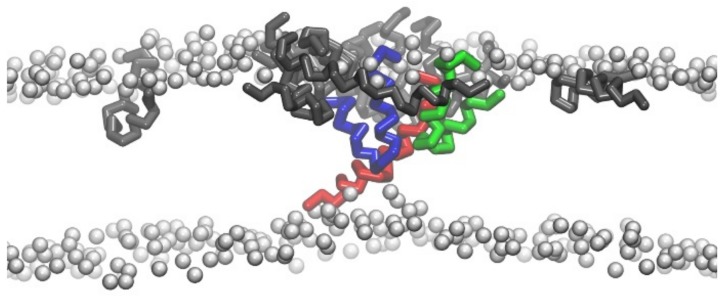
Aggregation and conformational change of melittin during insertion into a DLPC membrane. Peptides have various conformations and states, including U-shaped conformation (blue and green) and transmembrane insertion state (red). The other peptides are shown in black, while the lipid headgroups are shown as silver-grey beads. For clarity, the lipid tails are not shown.

It has been shown in simulations that peptide aggregation is always associated with the membrane insertion process of melittin. Such aggregation behavior also occurs in a concentration-dependent manner. For example, at a P/L ratio below 1/43, melittin mostly stays on the membrane surface in a monomer form; however, with an increase in P/L ratio, melittin starts to aggregate into clusters [78]. Importantly, it was observed that peptide aggregation was crucial for the reorientation and insertion of melittin (Figure 3). Sengupta et al. reported that formation of aggregates involving at least three peptide molecules was necessary for the insertion of melittin [40]. This claim was supported by the simulation results of Woo et al. [78] and Santo et al. [75]. However, it is worth noting that, based on previous simulation observations, not all the peptides aggregate into one cluster during their membrane action process; instead, they distribute unevenly on the membrane surface and have various aggregation forms with different inter-peptide association states. The residue–residue interactions are assumed to be key for such inter-peptide associations. Moreover, these associations are significantly influenced by the chain configuration and molecular orientation of melittin [40]. To sum up, although it was previously accepted that the P/L ratio was the most important for pore formation in a membrane by melittin, current simulations further revealed that the local concentration and distribution of peptides on a membrane, instead of the general P/L ratio of the whole system, were crucial in determining the membrane activities, e.g., insertion of melittin [40].

### 2.4. Conformational Changes of Peptide during Membrane Insertion

The interfacial interactions between membrane and peptides induce conformational changes in melittin. One representative example of such changes is the formation of α-helical structure of the membrane-bound melittin from a random-coiled state in solvents. It was argued that such a conformational change would make the hydrophobic and hydrophilic residues organized on the opposite faces of the helix, leading to the formation of amphiphilic structures to facilitate the membrane actions (e.g., insertion) of peptides [80,81]. In addition, it was interesting to observe that the peptide chain of melittin adopted a “U-shaped” conformation on/in the membranes during the membrane binding stage and even during the inserting process (Figure 2 and Figure 3, also see Supplementary Materials Movie S1). Note that the U-shaped melittin was generally observed during its interaction with membranes, even with different force fields (CG or AA) or lipid environments (e.g., POPC (1-palmitoyl-2-oleoyl-glycero-3-phosphocholine), DPPC, or DPPC/POPG) [54,75,76,78,82]. In particular, it was believed that U-shaped melittin might work as a wedge to facilitate peptide insertion and membrane poration [75,76]. However, to the best of our knowledge, such a “U-shaped” conformation of melittin has not been directly observed in experiments. More efforts are still needed to understand the influences of conformational changes on the membrane insertion process of melittin.

### 2.5. Structural Disturbance to Membrane during Melittin Insertion

Peptide action causes significant structural disturbance to lipid membrane, which is considered essential for realizing the transmembrane insertion of melittin [32,36,37]. Consistent with experimental observations, simulations also demonstrated obvious expanding and thinning of membranes under the influence of melittin adsorption/binding. These changes are dependent on both the P/L ratio and lipid types. As shown by Santo et al., for a DPPC/POPG bilayer, with a low P/L ratio of about ~1/100, the increase in area per lipid (APL) was less than 3%, and the decrease in membrane thickness (MT) was below 1%; in contrast, with an increase of P/L to 1/21, the APL increased by almost 12%, and MT decreased by 13% [75]. Similar changes were also observed for a DPPC bilayer in that, at a P/L ratio of 1/32, the APL was found to increase by 14%, and MT decreased by 10% [83]. More importantly, the expanding and thinning behaviors due to melittin actions are not symmetric between two leaflets across the membrane [40,83]. For example, Sengupta et al. found that the thickness fluctuation of the outer monolayer on which melittin adsorbed was stronger than that of the inner monolayer [40]. Such an asymmetry between the two leaflets would produce a profound influence on the membrane poration behavior of melittin, which will be further discussed later.

Changes in mechanical state of a membrane always accompany its structural changes. For example, under the actions of melittin, the distribution of membrane stress is changed notably. Considering that the structure of a lipid bilayer is planar, the membrane stress σ (σ_outer_ or σ_inner_ refers to the stress of the corresponding monolayer) can be calculated as follows:

(1) $$\sigma =\sigma_{outer}\left(Z\right)+\sigma_{inner}\left(Z\right)=-\int_{Z_{1}}^{Z_{0}}P\left(Z\right)dz+\int_{Z_{2}}^{Z_{0}}P\left(Z\right)dz$$

(2) $$P\left(Z\right)=P_{L}\left(Z\right)-P_{N}\left(Z\right)$$

(3) $$P_{L}\left(Z\right)=\left(P_{xx}+P_{yy}\right)/2$$

where *P_L_*(Z) is the local lateral pressure depending on the Z-coordinate, *P_N_*(Z) is the local normal pressure, and Z_0_ is the Z coordinate of the bilayer center, while Z_1_ and Z_2_ refer to positions outside of the two monolayers, respectively [84]. For an undisturbed lipid bilayer, the pressure distribution across the membrane is symmetric, that is, σ_outer_(Z) ≈ −σ_inner_(Z) or σ=0. However, under the actions of the membrane-bound melittin, the symmetry of the pressure is broken. Goliaei et al. drew the Z-dependent distribution profiles of membrane pressure both before and after peptide binding [84]; a hump appeared in the region corresponding to lipid tails of the outer monolayer (exactly the peptide binding site), while a decrease occurred simultaneously in the tail region of the inner monolayer. Quantitative analysis demonstrated that the stress of each monolayer changed differently, as σ_outer_ = −6.4 ± 0.2 mN/m, σ_inner_=6.9 ± 0.8 mN/m (when P/L = 1/50) or σ_outer_ = −8.8 ± 0.3 mN/m, σ_inner_ = 9.4 ± 0.8 mN/m (when P/L = 1/33). The opposite signs of σ_outer_ and σ_inner_ indicated forces in opposite directions experienced by the two monolayers. Moreover, the inequality in the magnitudes of the stresses (|σ_outer_| ≠ |σ_inner_|) finally caused membrane bending and facilitated the insertion of peptides.

**Figure 4:**
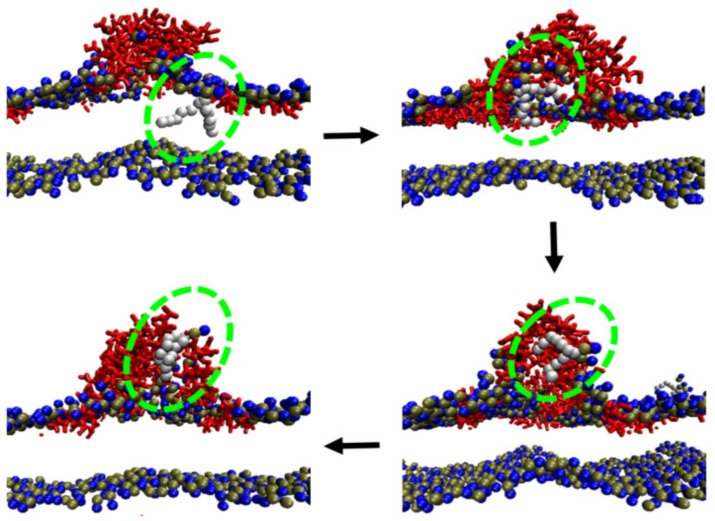
A typical structural disturbance to membrane by melittin: lipid extraction. The extraction process of a lipid (with tails in silver-grey and headgroup in blue and dark green) from a DOPC bilayer by melittin (red) is highlighted with a green dashed circle and indicated with black arrows. Lipid headgroups are displayed with blue and dark green spheres. Lipid tails are not shown for clarity. The figure is reprinted with permission from [85], copyright 2018 Elsevier.

Actually, membrane deformations have been widely observed in simulations [75,76,85,86], and the deforming degree was found to increase with an increase in the P/L ratio [75,85]. Moreover, an interesting behavior, lipid extraction, which normally occurs at a highly deformed membrane region, was observed in simulations [85,87]. Lipid extraction is an indication of the complicated melittin–membrane interactions. It generally occurs due to peptide aggregation on/in the membrane and the resultant strong membrane deformation [85]. Under the influences of the aggregated peptides, including relocation or partial insertion of melittin on/in the membrane and the consequent perturbation to lipid arrangements [87], lipid molecules are extracted from the membrane (and even totally pulled out) leading to the formation of a lipid–peptide complex (Figure 4). More interestingly, Liu et al. found that lipid extraction occurred exclusively on the outer leaflet of the bilayer (Figure 4). This manner of interaction between melittin and membranes was further confirmed in experiments via photoluminescence and dissipative quartz crystal microbalance (QCM-D) tests using supported lipid bilayer models [85]. Selective extraction of lipids from the outer leaflet induced an asymmetric distribution in lipid numbers between the two leaflets, and consequently, the pressure distribution across the membrane was altered notably. Thus, the free-energy barrier for the reorientation and insertion of melittin peptides into membranes decreased significantly. Accordingly, transmembrane pores with a torus-like shape were found to be formed near the accumulated peptide region of an asymmetric lipid membrane [85]. In addition, in the simulations by Lafleur and coworkers, melittin was found to extract 1,2-dipalmitoyl-sn-glycero-3-phosphocholine (DPPC) and 1,2-Dipalmitoyl-sn-glycero-3-phosphoserine (DPPS) lipids with the same proportions from a binary DPPC/DPPS membrane [80], which means melittin has no preference between DPPC or DPPS during the extraction process. However, for another lipid membrane system, melittin was found to selectively extract phosphatidylcholines from phosphatidylcholine/phosphatidylethanolamine membranes [88]. These contradictory observations indicate the complexity of melittin–membrane interactions, which further influence the packing states of lipids and insertion manners of peptides [88].

## 3. Thermodynamic Analysis of Transmembrane Insertion of Melittin: Decreased Free-Energy Barrier due to Inter-Peptide Cooperation

Transmembrane penetration of melittin is difficult to occur due to the high free-energy barrier required for the reorientation and insertion process of peptides. Therefore, cooperation between peptide molecules is of much significance [39,83]. Thermodynamic analysis is undoubtedly helpful to give deep insights into the underlying physics of the cooperative effect between peptides.

### 3.1. Potential of Mean Force (PMF) Distribution Demonstrating the Inter-Peptide Cooperation

**Figure 5:**
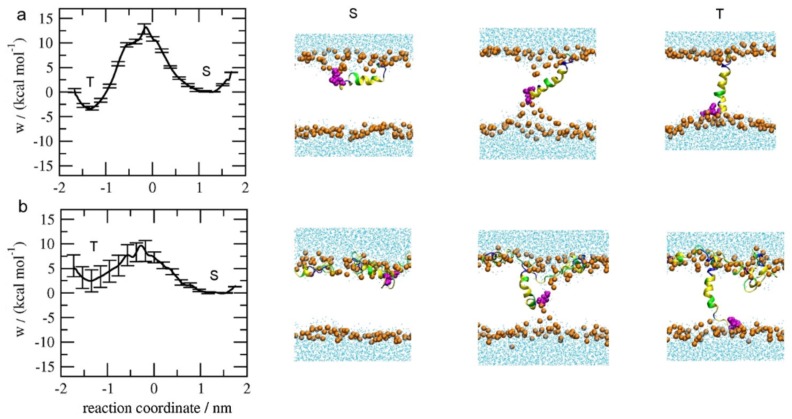
Potential of mean force (PMF) distribution during insertion of the first peptide into membranes with different peptide concentrations. (**a**) P/L=1/128; (**b**) P/L=4/128. The first three residues at the N-terminus are colored in magenta. The figure is reprinted with permission from [89], copyright 2013, American Chemical Society.

The transmembrane insertion of melittin could be further subdivided into two cases: the insertion of the first peptide molecule into the membrane, and the subsequent insertion of other peptides into the membrane with embedded peptides. In both cases, inter-peptide cooperation plays important roles. Irudayam and coworkers provided the free-energy profiles for the transition of a melittin peptide from a membrane-binding state (i.e., *S* state) into a transmembrane-inserting state (i.e., *T* state) under various P/L ratios with a POPC bilayer, by calculating the potential of mean force distributions with the weighted histogram analysis method (WHAM) [89]. As shown in Figure 5, with a P/L ratio of 1/128, there was only one melittin in the system, and the free-energy barrier for the transition of melittin, i.e., from *S* state to *T* state, was around 13.20±0.8 kcal/mol. However, with a higher P/L ratio of 4/128, the energy barrier decreased to 9.6±1.9 kcal/mol. In this case, peptide aggregation was observed near the inserted melittin, and the membrane structure was also disturbed. In addition, the POPC bilayer was found to be stretched by 9.3% at the higher P/L ratio. Furthermore, many featured behaviors, such as partial folding and unfolding of the peptide, as well as formation of the U-shaped peptide configuration, were observed during the peptide insertion process. These phenomena indicate a possible contribution of the peptide conformational entropy to the free-energy change during the reorientation of melittin from the *S* to *T* state [89]. In general, it has been demonstrated that cooperation between peptides is helpful for lowering the free-energy barrier during membrane insertion of melittin, although the detailed cooperation manners are still unknown.

**Figure 6:**
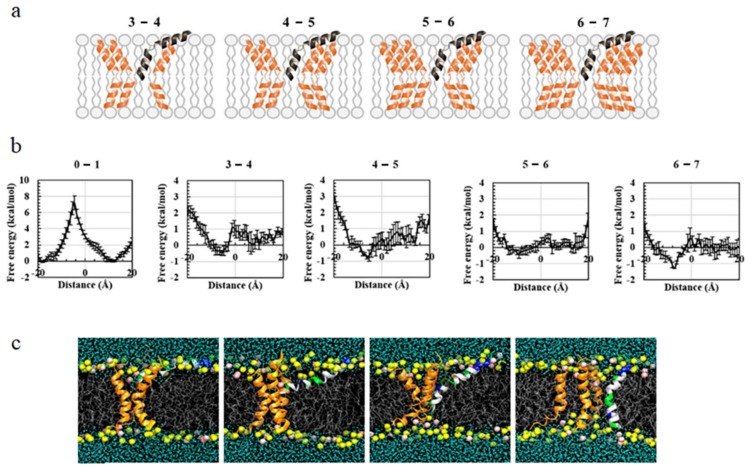
PMF distribution during insertion of melittin into a membrane with embedded peptides. (**a**) Sketches showing the insertion of a peptide (black) into a pre-existing pore of different sizes; (**b**) PMF profiles under various situations; (**c**) snapshots showing the translocation of a single peptide into a pore consisting of a three-peptide aggregate. From left to right, the peptide inserting position is: 11.99 Å, 4.49 Å, −6.21 Å, and −15.34 Å. The figure is adapted with permission from [91], copyright 2017, AIP Publishing.

During the second case in which melittin inserts into a membrane with embedded peptides, cooperation also takes effect. With an increase in the embedded peptide number, situations become even more complicated [90]. To understand this issue, Lyu et al. analyzed the PMF distributions during the insertion of melittin into a DOPC/DOPG mixed bilayer containing three to six transmembrane peptides, respectively (Figure 6) [91]. The free-energy barrier for the first insertion was high, with a large value of around ~7.209±0.864 kcal/mol being in agreement with the results of Irudayam et al. [89]. However, the following insertion became easier, and the barrier value decreased with an increase in the number of embedded peptides. For example, when six peptides are in the membrane, the barrier for the insertion of the seventh melittin was only 0.588±0.185 kcal/mol [91]. Furthermore, more than one barrier was observed in the melittin insertion process. For example, during the fourth insertion of melittin, two barriers were found at the Z positions of 4.49 Å and −1.04 Å, respectively. The former one might be attributed to the partial insertion of hydrophobic residue and consequent bending of the upper lipid head. The latter one might be due to the further insertion of charged residues (e.g., 7 Lysine) and further bending of lipid heads. Note that the existence of multiple transmembrane positions during the transferring process of peptides, i.e., the upper leaflet, the center, and the lower leaflet, in addition to the surface and bottom of the bilayer, were also observed in single molecular experiments [92]. Moreover, it was observed that the PMF profile throughout the transmembrane insertion process of a melittin peptide was not symmetric, which was mostly ascribed to the deviations in lipid packing states and the deformation of the membrane. In addition, the property and distribution of key residues were found to significantly determine the peptide insertion behavior. In general, melittin experiences rather complicated environment changes during its interaction process with the membrane. Many factors, including the lipid packing states, membrane deformation degree, and key residues’ properties of the peptide, may contribute to the free-energy profiles during the membrane insertion of melittin. Therefore, a full description of the multi-dimensional free-energy surface with proper reaction coordinates is necessary for an in-depth understanding of the insertion behavior of this AMP.

### 3.2. Influence of Lipid State on Free-Energy Barrier during Peptide Insertion

The states of lipids significantly affect the behavior of peptides in a membrane. Hu et al. made a clear correlation between lipid states and pore-forming thermodynamics by using MD methods [93]. They found that the influences of many structural parameters of lipids, including the tail-chain length, saturation degree, and headgroup types, were all coupled together in determining peptide behavior. For the lipids with equivalent headgroup types and tail saturation states but different tail chain lengths (reflected by the number of beads in coarse-grained lipid models), they found that $\Delta G_{pore}$, i.e., the free energy to create a 3-nm-radius pore, increased linearly with the increase of tail length, as DHPC (1,2-dihexanoyl-sn-glycero-3-phosphocholine, 8 beads) < DLPC (10 beads) < DPPC (12 beads) < DSPC (14 beads). The enhanced barriers might be associated with the strengthened bending rigidity for corresponding lipid membranes ($\kappa_{c}=5.89,\;8.97,\;11.59,\;13.59\times 10^{-20}J$ for DHPC, DLPC, DPPC, and DSPC, respectively). In contrast, for the bilayers with a similar thickness but different tail saturation degrees, the free-energy barrier was found to be positively correlated with APL. However, when the lipids had identical tail-groups but different headgroups, the cost of free energy was anti-correlated with APL.

## 4. Conclusions and Perspectives

**Figure 7:**
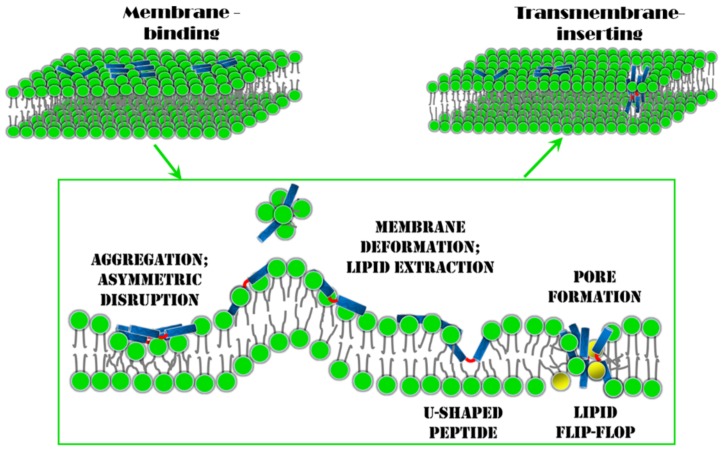
Diagrammatic sketch showing the membrane insertion process of melittin. Between the initial membrane-binding and final transmembrane-inserting states, aggregation and conformational changes occur to the peptides, leading to asymmetric disruption and lipid extraction of the membrane. All these issues are regarded as key factors determining the membrane insertion of melittin. Melittin is represented with two blue rods connected by a red line. Lipids are shown with green heads and dark grey tails.

The membrane insertion behavior of melittin is a key step for its pore-forming and antibacterial activities, while it is also the most complicated step in its interaction with the membrane (Figure 7). Computer simulations have unique advantages in exploring the molecular details of this process and in revealing the underlying mechanism [48,94]. Based on current simulation results, to overcome the high free-energy barrier for the transition of melittin from a membrane-binding state into a transmembrane-inserting state, the aggregation of the peptides on/in the membranes, which provides the possibility for inter-peptide cooperation, is crucial (Figure 7). Under the action of aggregated melittin, the membrane’s structure is disturbed in an asymmetric manner between the two leaflets. Accordingly, unequal stress is produced in the two monolayers, and the mechanical state of the membrane is strongly altered. Consequently, thinning and expansion occur in the membrane, along with strong fluctuations in local curvature. Moreover, some lipids would be extracted exclusively from the outer leaflet, leading to asymmetry in the lipid number between the two leaflets (Figure 7). All these changes collaboratively reduce the free-energy barrier for melittin transition and facilitate the final insertion of peptides into membranes. On the other hand, with the translocation of melittin into the membrane, the peptide may deform itself into a “U-shaped” conformation as an intermediate transition state, and lipids around it would bend or incline themselves accordingly (Figure 7). The sophisticated intermolecular interactions between lipid–peptide, and even inter-peptide, significantly influence the detailed membrane insertion process of melittin. Thus, melittin would experience multiple energy barriers throughout the insertion process, and the membrane structure would be further changed (e.g., lipid bending or flip-flop, Figure 7).

For natural melittin, a high P/L ratio is normally necessary to overcome the high free-energy barrier for its membrane insertion. This indicates poor antimicrobial efficiency when the concentration of melittin is low, which severely hinders its clinical applications. Based on the discussions above, two strategies are especially promising to accelerate the membrane insertion of melittin and facilitate its biomedical applications.

One is to design the amino acid sequence of pristine melittin by taking into consideration its membrane interaction mechanism to improve its membrane insertion efficiency. Recently, Wimley and coworkers have made notable experimental progress with respect to the enhancement of the membrane activity of melittin via residue mutations [95,96,97,98,99]. By using an orthogonal high-throughput assay, about a dozen sequences were screened from a 7800-member library, which differed from the parent melittin in only a few amino acids out of the total 26 but were highly potent in membrane poration. In particular, as the most active gain-of-function variant with five-site mutation (i.e., T10A, R22A, K23A, R24Q, and Q26L), MelP5 could make stable transmembrane pores in lipid vesicles at an extremely low P/L ratio (P/L≤1/1000) which was approximately 20-fold lower than that required for the native melittin. Future development in designing high-performance AMPs depends on our understanding of the sequence–structure–function relationship of melittin or other AMPs. In simulations, the combination of kinetic and thermodynamic descriptions of melittin–membrane interactions, such as the calculations of high-dimensional free-energy surfaces with proper reaction coordinates chosen from corresponding kinetic processes, would be helpful to decouple the correlated influential factors for melittin insertion and unearth this important relationship [73]. New calculating methods and theoretical approaches [100,101,102], such as machine learning, would also make significant contributions [103,104,105].

To engineer melittin with nanomaterials is also an intriguing pathway to improve the performance of melittin. It has been shown that the bacteria-killing efficacy of AMPs could be improved by decorating the peptides on the nanoparticle surface or self-assembling the AMP-based derivatives into nanostructures [41,106,107,108,109,110,111,112,113,114,115]. The improved bacteria-killing ability is mostly induced by the increased local peptide concentration of AMPs, which strengthens the potential cooperation between peptides. Very recently, the in silico design of a hybrid complex composed of melittin and graphene materials (e.g., graphene or graphene oxide nanosheets) was reported [116]. Simulations and experiments showed that the as-fabricated complex exhibited remarkable efficiency in transmembrane perforation due to the synergetic insertion of graphene materials and melittin into membranes. The complex demonstrated a poration velocity that was two orders of magnitude faster than the pure melittin, an over 10-fold decrease in the threshold working concentration of peptide, and consequently an up to 20-fold enhancement in antibacterial activity against both Gram-negative and Gram-positive bacteria. Promisingly, the combination of nanotechnology and biotechnology will greatly benefit the biomedical applications of melittin or other AMPs.

## Supplementary Materials

The following are available online at https://www.mdpi.com/1420-3049/24/9/1775/s1, Video S1: Formation process of U-configuration and the transmembrane penetration of melittin.
